# Response to Sun and Colleagues: Charting a Simpler Ethical Landscape of Generative AI‐Augmented Clinical Documentation

**DOI:** 10.1111/jep.70232

**Published:** 2025-07-28

**Authors:** Richard C. Armitage

**Affiliations:** ^1^ School of Medicine, Academic Unit of Population and Lifespan Sciences, Clinical Sciences Building, Nottingham City Hospital Campus University of Nottingham Nottingham UK

## 1 Introduction

The recent article by Sun and colleagues sets out to identify key ethical considerations for the use of generative AI (artificial intelligence) in clinical documentation and offers recommendations to address these concerns [1]. Health equity considerations, the clinician–patient relationship, and algorithmic transparency and integrity are identified as key ethical considerations, and focusing on enhancing patient autonomy, ensuring accountability, and promoting health equity are suggested to mitigate these concerns.

At the outset of the article, the scope of the enquiry is set as focusing ‘on the use of generative AI chatbots for clinical documentation.’ It is clarified that generative AI‐assisted clinical documentation refers to ‘the process of summarising patient interactions into encounter notes or handoff reports, drafting discharge or after‐visit summaries, generating supporting documents for processes such as prior authorisations, and related documents, but without independently initiating clinical decisions’.

Accordingly, the use of generative AI for the purpose of clinical documentation production within this scope would only extend to the summarisation of two kinds of source material: first, clinician‐written clinical notes, such as admission notes for the production of handoff reports or discharge summaries; second, patient–clinician verbal discussions, such as for the generation of after‐visit summaries or prior authorisation documents.^1^ In both kinds, the source material is generated by the clinician alone or by the clinician and patient, and not by the generative AI. The generated documentation would be used by clinicians (such as the same doctor reading after‐visit summaries at a later date, or other clinicians like hospital doctors reading handoff reports or primary care doctors reading discharge summaries), insurers (such as prior authorisation documents), and patients (such as lay language discharge summaries).

The article correctly identifies three relevant ethical considerations regarding the use of generative AI in broader clinical practice. However, these arise from the deployment of generative AI beyond the stated scope of the article. If, instead, the enquiry remains within its stated scope—‘the use of generative AI chatbots for clinical documentation’, which involves summarising clinician‐written clinical notes or patient–clinician verbal discussions—the identified ethical considerations either do not arise or are easily mitigated by AI‐generated clinical documents being reviewed, edited, and approved by the clinician using these tools. This shall now be explained in the same order as addressed in the article.

## 2 Ethical Considerations of AI‐Generated Clinical Documentation

### 2.1 Health Equity Considerations

The article is concerned that ‘generative AI systems have embedded biases that exacerbate inequities in generated content.’ It discusses representation bias in which ‘underrepresented groups in training data yield less accurate outputs for certain groups’, and cites ‘lower performance in pulse oximetry reading or skin lesion identification on darker skin tones,’ ‘underestimation of cancer incidence in under‐resourced communities’, ‘[the] requirement of greater disease burden for black patients to be recommended for care than white patients’, and ‘exaggerating stereotypical demographic presentations of illnesses in generated text’ as examples. However, the first three of these four examples do not involve generative AI, and none refer to the scope of the article—‘the use of generative AI chatbots for clinical documentation’ (the only example that uses generative AI—the fourth—examined the potential applications of a kind of generative AI in medical education, diagnostic reasoning, clinical plan generation, and subjective patient assessment) [2]. While these are important ethical considerations for the use of generative AI in broader clinical practice, they do not apply to the use of these technologies for summarising clinician‐written notes or patient–clinician verbal discussions to produce clinical documentation.

Accordingly, the biases that the article worries might appear in AI‐generated documentation, such as that which ‘may misgender patients,’ would only occur if the same biases were contained within the source material summarised by the generative AI. This would not be the product of generative AI, but of the clinician‐written clinical notes or patient–clinician verbal discussions. As such, the only biases that ‘may propagate across multidisciplinary care teams’ via clinical documentation are those that originated within the source material, and not within the AI‐generated documentation.

### 2.2 Clinician–Patient Relationship

The article worries that AI‐generated clinical documentation might negatively impact the clinician–patient relationship. For example, ‘documents generated by AI when the clinician is not the primary author may de‐emphasise the most salient points of the visit’. However, while the clinician might not be the document's primary author, they will be the document's reviewer, editor, and final approver, meaning the most salient points of the visit (which was conducted by the same clinician) will not be de‐emphasised. Similarly, the concern that AI‐generated summaries might not capture the nuances in ethically sensitive discussions, such as the use of overly deterministic rather than conditional language, is dealt with by the clinician using the tool editing the AI‐generated document to reflect these nuances.

### 2.3 Algorithmic Transparency and Integrity

The article is concerned that ‘the intrinsic opacity of complex AI decision‐making processes… creates an unresolved void in accountability’ and this ‘lack of transparency is compounded by the fact that generative AI models may generate flawed rationales in clinical reasoning’. Yet, the scope of the article is ‘the use of generative AI chatbots for clinical documentation’, not for clinical reasoning or any other feature of clinical practice. Any concerns of transparency regarding AI‐generated summaries of clinician‐written clinical notes or patient–clinician verbal discussions can be dealt with by the clinician using these tools having access to the summaries’ source materials for cross‐referencing.

As such, within the stated scope of the article, the identified ethical considerations either do not arise or are easily mitigated by the clinician using the generative AI. Accordingly, beyond the requirement for AI‐generated clinical documents to be reviewed, edited, and approved by the clinician using these tools (which the article recommends), the proposed recommendations are unnecessary. This shall now be explained in the same order as addressed in the article.

## 3 Proposed Recommendations for AI‐Generated Clinical Documentation

### 3.1 Patient Autonomy

The article proposes ‘early adoption of transparency measures by including patient notifications when AI‐generated documentation may meaningfully impact their understanding of care…. [including] cases where AI generates after‐visit or discharge summaries’. However, since ‘harm and bias in documentation’ do not arise when generative AI is used within the scope of the article and edited by the clinician, patient autonomy is not obstructed, thereby rendering such transparency measures unnecessary.

Furthermore, the article recommends that the ‘raw data [or source material] underlying AI‐generated documentation, such as transcripts of clinician–patient discussions, should be retained for a specified period to allow validation of AI‐generated summaries by clinicians or patients’. However, the first kind of source material (clinician‐written clinical notes) are already routinely retained, meaning no change in practice is required here. The second kind of source material (patient–clinician verbal discussions) is not routinely retained because records of it (transcribed discussions) are not routinely collected. Since clinician‐written after‐visit summaries are routinely retained without a transcription of the underlying patient–clinician verbal discussion being collected, the transcribed discussions with which generative AI produces the after‐visit summaries that are reviewed, edited, and approved by the clinicians conducting the visits need not be retained. Accordingly, the article's recommendation is unnecessary, and its associated administrative, financial, and data security burdens can be avoided.

### 3.2 Accountability

The article identifies ambiguities over accountability of documents produced by generative AI and recommends that ‘measures should address liability concerns for when AI causes harm to patients’. However, the use of generative AI within the stated scope of the article does not raise such ambiguities, because the clinician using generative AI tools remains accountable for the AI‐generated documents they review, edit, and approve in the same way they are accountable for the documents they produce without generative AI assistance. Similarly, the article is concerned about variation in the degree to which language in AI‐generated documents to be read by patients is simplified by the patient's race/ethnicity (this concern emerged from the performance of retired generative AI models that have since been replaced by multiple improved iterations) [3]. However, this concern is also unnecessary, because the clinician who both knows the patient and uses the generative AI tools can themselves edit the AI‐generated document to meet the individual patient's literacy ability before approving the document.

### 3.3 Health Equity

Finally, the article recommends that ‘training data [must] represent the population they are intended to serve, including historically underrepresented groups, by sourcing data from multiple regions, healthcare settings and socioeconomic strata’, and ‘the importance of incorporating social determinants in these models’. While these are pertinent to the use of generative AI in broader clinical practice, they are not relevant to the use of generative AI within the stated scope of the article, which simply involves the summarisation of clinician‐written clinical notes or patient–clinician verbal discussions.

In summary, the article identifies ethical considerations that, within the stated scope of the article, either do not arise or are easily mitigated by AI‐generated clinical documents being reviewed, edited, and approved by the clinician using these tools. Accordingly, the article's recommendations are largely unnecessary, and their associated administrative, financial, and data security burdens can be avoided.

## Conflicts of Interest

The author declares no conflicts of interest.

## Data Availability

Data sharing is not applicable to this article, as no new data were created or analyzed in this study.

## Endnotes

1 The first kind requires the technology to ‘understand’ the text it ‘reads’; the second kind requires the technology to ‘understand’ the text, once text has been generated by automated speech recognition technology, it ‘reads’; and both require the technology to summarise this text.
